# Global Remote Sensing Research: An Author‐Centric Analysis of Contributions and Trends

**DOI:** 10.1002/ece3.71952

**Published:** 2025-08-08

**Authors:** Ehsan Rahimi, Chuleui Jung

**Affiliations:** ^1^ Agricultural Science and Technology Institute GyeongKuk National University Andong Republic of Korea; ^2^ Department of Plant Medicals GyeongKuk National University Andong Republic of Korea

**Keywords:** citation analysis, climate change, forests, google scholar, network analysis, trend analysis

## Abstract

Remote sensing research stands at a critical juncture, grappling with challenges in identifying shifting trends and emerging scientific priorities. These gaps limit the field's ability to effectively address pressing environmental issues and foster innovation in related applications, particularly in conservation. In this study, we conducted a comprehensive scientometric analysis of approximately 20,000 researchers identified via Google Scholar who explicitly list “remote sensing” in their profiles, examining their publication trends, citation patterns, collaboration networks, and disciplinary affiliations. The analysis utilized a dataset comprising over 837,658 publications and nearly 20 million citations spanning from 1700 to 2024. Our findings reveal that each researcher has accumulated an average of 1435 citations, with a mean H‐index of 10.9 and an i10‐index of 17.4. Collaboration plays a pivotal role in the field, as evidenced by 79% of citations originating from co‐authored works. The peak of scientific output was observed in 2022, with 54,304 publications—the highest annual total recorded. However, a slight decline was noted in 2024, with 50,096 papers published. Citations per paper peaked in the mid‐2010s, reaching their highest levels between 2015 and 2020. In contrast, recent years have witnessed a marked decline, with total citations dropping from over 1.1 million in 2020 to 317,585 in 2023 and just 84,389 in 2024. Keyword analysis identified “classification,” “climate,” “forest,” “land,” and “mapping” as dominant themes, reflecting the field's continued focus on addressing global environmental challenges. While the sustained growth in publication output underscores the dynamism of remote sensing research, the declining citation counts suggest a shift toward highly specialized studies that appeal to narrower audiences. This trend necessitates a strategic reassessment of research priorities and publishing practices to ensure that remote sensing studies maintain their relevance and impact in addressing urgent global challenges.

## Graphical abstract

Our study analyzes publication and citation trends among ~20,000 remote sensing researchers using a dataset of over 837,000 publications and 20 million citations. Results reveal peak scientific output in 2022 and a sharp citation decline by 2024, highlighting a shift toward narrower, specialized research. The findings underscore the need for strategic realignment in remote sensing to sustain impact on global environmental challenges.

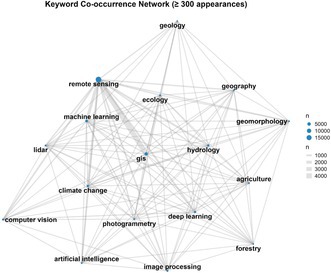

## 1 Introduction

A close examination of the literature highlights extensive bibliometric investigations across various topics. For example, ecosystem services have been a significant focus, with several studies analyzing their trends and implications (Vihervaara et al. 2010; Liu et al. 2019; Zhang, Estoque, et al. 2019; Gangahagedara et al. 2021; Wang et al. 2021; Chen et al. 2022; Tasneem and Ahsan 2024). Similarly, the impact of climate warming has garnered extensive attention, leading to substantial bibliometric research aimed at understanding its multifaceted effects on ecosystems (Bjurström and Polk 2011; Haunschild et al. 2016; Deng et al. 2017; Marx et al. 2017, 2021; Sweileh 2020; Das et al. 2021; Li, Zheng, and Zhang 2021; Nalau and Verrall 2021; Fan et al. 2022; Liang et al. 2023; Setiawan et al. 2023; Jiang et al. 2024; Li et al. 2024; Lindawati and Meiryani 2024; Pius Awhari et al. 2024). Beyond these topics, urban wildlife ecology and conservation (Collins et al. 2021), deforestation (Aleixandre‐Benavent et al. 2018; Valjarević et al. 2018), ecological restoration (Zhang et al. 2010; Wei et al. 2022; Zheng et al. 2023; Shi et al. 2024), movement ecology (Holyoak et al. 2008), and ecological infrastructure (Sun et al. 2020) have also emerged as areas of considerable scholarly interest.

In this regard, remote sensing (RS) has emerged as a transformative tool across diverse scientific disciplines, driving innovation in ecological monitoring, environmental management, and technological advancement (Rahimi et al. 2025). Over the years, bibliometric analyses have offered valuable insights into the thematic trends and applications of remote sensing, highlighting its growing relevance in addressing pressing global challenges (Zhang et al. 2017; Santos et al. 2021). These studies provide a comprehensive understanding of research priorities, geographic contributions, and technological advancements, yet they often overlook the critical roles played by individual researchers and collaborative networks in shaping the field (Rahimi and Jung 2025). Thematic analyses reveal significant progress in remote sensing applications, such as monitoring soil organic matter (SOM) and forest biomass. Since 2018, advancements in modeling techniques, data preprocessing methods, and international collaboration have driven exponential growth in RS‐based SOM research (Chen et al. 2025). Similarly, remote sensing has become indispensable for accurately monitoring forest biomass, a critical factor in understanding carbon dynamics and achieving global carbon neutrality. An analysis of 1678 studies from 1985 to 2023 highlighted an average annual growth rate of 2.64%, emphasizing the expanding interest in this area (Shi et al. 2024).

Geographically, remote sensing research exhibits diverse patterns of growth and contribution. For example, RS research in Vietnam experienced steady growth from 2000 to 2019, with rapid acceleration after 2015, driven by key institutions focusing on topics like climate change, land‐use change, and machine learning applications (Pham‐Duc et al. 2020). In contrast, China, a leading contributor to global remote sensing research since the 1970s, has integrated RS into national economic and social development. A bibliometric analysis of 963 highly cited papers from 2010 to 2014 revealed advancements in platforms and sensors, with a focus on multi‐source data processing and geographic monitoring (Zeng et al. 2015). In a separate study focusing on global grassland remote sensing research, Li, Cui, et al. (2021) performed a scientometric analysis of 2692 publications and more than 82,000 references from 1980 to 2020. Their results showed a rapid increase in the number of publications, particularly after 2010, with remote sensing, environmental sciences, and ecology emerging as the primary research fields. Through keyword and co‐citation analyses, they identified seven key research clusters and 17 prospective research directions, emphasizing the potential of emerging technologies such as unmanned aerial systems, cloud computing, and deep learning to further advance grassland remote sensing in support of sustainable development goals.

The thematic evolution of RS applications is evident in specific fields such as mineral exploration, algal bloom monitoring, and land degradation in drylands. Remote sensing for mineral exploration has gained global significance, with a bibliometric analysis of 37,977 publications (2000–2022) highlighting extensive international collaboration and the development of advanced sensors (Ju et al. 2024). Similarly, studies on algal blooms reveal both advancements and limitations in RS applications, with research peaking in 2014 (Sebastiá‐Frasquet et al. 2020). In drylands, remote sensing research has surged since 2011, addressing critical challenges related to climate change and human activities, with a focus on land use and vegetation dynamics (Costa et al. 2023).

Technological innovation has further propelled the field. The use of satellites like Landsat, MODIS, and LiDAR has dominated RS applications, enabling advancements in change detection, biodiversity conservation, and climate change impact assessment (Duan et al. 2020). Emerging technologies, such as unmanned aerial vehicles (UAVs), small satellites, and machine learning algorithms, have expanded the scope of remote sensing, improving data acquisition, processing, and applications across disciplines (Zhang et al. 2017; Balz 2022). Platforms like Google Earth Engine (GEE) exemplify this growth, with nearly 85% of GEE‐related research published in the last 3 years, focusing on topics like land use, water resources, and climate change (Pham‐Duc et al. 2023).

Although numerous studies have examined trends in specific remote sensing areas—such as urban remote sensing (Wentz et al. 2014), UAV imagery for crop mapping (Nduku et al. 2023), soil moisture monitoring (Badaluddin et al. 2021), global NDVI research (Xu et al. 2022), future directions in remote sensing (Khorram et al. 2016), global vegetation status and changes (Li et al. 2023), advances in space‐borne optical remote sensing (Dash and Ogutu 2016), and global analyses of Google Earth Engine (Velastegui‐Montoya et al. 2023)—existing bibliometric analyses often lack a focus on individual researchers. This author‐centric perspective is crucial for understanding how researchers and their networks shape (Rahimi and Jung 2025) the progression of knowledge in remote sensing. Important metrics such as citation trajectories, H‐indices, and shifts in research topics remain insufficiently explored, leaving gaps in identifying the key drivers of innovation and collaboration (Hussain 2017; Borthakur and Singh 2018). Addressing these gaps requires an integrated approach that combines thematic trends with detailed author‐level analyses to better reveal the complex dynamics guiding the development of remote sensing research.

This study seeks to address these gaps by conducting a large‐scale, author‐centered scientometric analysis of remote sensing research, focusing on temporal patterns in publication trends, citations, collaborations, and disciplinary scope. To ensure conceptual clarity, we explicitly distinguish between satellite‐based detection approaches—which involve the acquisition and processing of spectral, spatial, and temporal data from remote sensing platforms—and machine learning techniques, which are increasingly used for data classification, modeling, and prediction within RS workflows. The literature reviewed spans from 1700 to 2024 and was sourced from publicly available Google Scholar profiles and academic databases, with a focus on researchers who self‐identify with “remote sensing” in their listed keywords.

## 2 Methods

### 2.1 Scholars' Profiles

To conduct an in‐depth analysis of experts specializing in remote sensing or employing its techniques across various disciplines, the first step involved assembling a comprehensive dataset of individuals actively engaged in the field. Google Scholar served as the primary resource for this task, offering an extensive database of academic profiles along with detailed bibliometric information. The platform provides critical indicators, including total citations, average citations per publication, H‐index, i10‐index, publication timelines, and a complete list of authored works. Researchers can also emphasize their expertise by listing up to five keywords in their profiles. By using the keyword “remote sensing,” we identified approximately 20,000 scholars whose profiles contained this term in English and systematically documented their profile URLs by late 2024.

While other platforms such as Scopus, Web of Science, SciProfiles, and ResearchGate also provide author‐level data, each has notable limitations. For example, Scopus does not index all publications, often resulting in lower citation counts compared to Google Scholar. Other platforms lack comprehensive citation metrics or omit researcher‐defined keywords entirely. Notably, neither Scopus nor Web of Science allows straightforward identification of researchers based on their declared research interests or keywords—such as “remote sensing”—at the profile level. In contrast, Google Scholar profiles often include user‐defined keywords, making it feasible to systematically identify researchers working in specific fields. Furthermore, these commercial databases do not provide openly accessible APIs or compatible R/Python packages for large‐scale data retrieval, which limits their utility for this type of study. For these reasons, we relied exclusively on Google Scholar, which allowed us to efficiently identify relevant profiles and extract consistent bibliometric information for analysis. Furthermore, our analysis was constrained by the availability of reliable R and Python packages that support large‐scale, automated extraction of researcher profiles. Given these practical considerations, we relied exclusively on Google Scholar and selected widely used metrics—total citations, H‐index, and i10‐index—to ensure consistency and comparability across the dataset.

The R package “Scholar” (available on CRAN: Package scholar) was instrumental in extracting this data. It allowed for the collection of bibliometric metrics from Google Scholar profiles, including total citations, H‐index, i10‐index, and a detailed breakdown of their publications. These publications were categorized by year, citation count, and authorship details. Additionally, we retrieved up to five keywords from each researcher's profile to identify their key research priorities. To enrich the analysis, we calculated averages for total citations, H‐index, and i10‐index across the dataset. Furthermore, we assessed the influence of collaborative work by comparing citation data from articles where the researcher was the first author to those where they were listed as co‐authors. This analysis highlighted the role of collaboration in shaping individual research impact and advancing the field of remote sensing.

### 2.2 Keyword Network Visualization

Our analysis also centered on uncovering distinct clusters of remote sensing scientists by examining the keywords featured in their Google Scholar profiles. To reveal these patterns, we employed keyword network visualization, which provided a detailed overview of the interconnected themes reflected in the titles of their publications. This approach highlighted their primary focus on remote sensing while also illuminating secondary areas of interest within the broader scientific landscape.

### 2.3 Trends Analysis for All Papers and Citations

We conducted a thorough assessment of all publications and their corresponding citation records for each remote sensing researcher, covering the period from 1960 to 2024. This analysis enabled us to identify yearly patterns in publication activity and citation rates, offering a clearer view of how the field has evolved and expanded over time. By tracking these trends, we uncovered valuable insights into the growth and development of remote sensing as a scientific discipline. To further explore thematic priorities within the field, we analyzed the most frequently appearing keywords in article titles, alongside the corresponding citation metrics. This involved identifying the five most commonly used terms and evaluating their citation impact. This preliminary investigation highlighted the dominant research topics and their relative significance over time, shedding light on key areas of focus within the community. Recognizing that keyword frequency alone might not fully capture the nuances of changing research priorities, we extended our analysis with more advanced methodologies to better understand the complexity and dynamic nature of research trends in remote sensing.

## 3 Results

### 3.1 Summary of Scholar Profiles

A comprehensive analysis of Google Scholar data for 19,826 remote sensing scientists offers valuable insights into their academic productivity, research impact, and collaborative efforts. After eliminating duplicate records, the dataset comprises 837,658 unique publications, which collectively have garnered 19,852,700 citations. On average, each researcher has accumulated 1435 citations. However, a substantial standard deviation of 5768 indicates significant variability, with some scientists achieving exceptionally high citation counts while others maintain more moderate levels of recognition (Table 1).

**TABLE 1:** The mean of total citation, H index, i10 index, mean total citation for authors as first author, and mean percentage of citations based on collaboration.

	Mean total citation	H‐Index	i10‐index	Mean total citation as first author	Collaboration proportion %
Mean	1435	10.9	17.4	290	79%
SD	5768	13.4	41.3	1122	

The average H‐index of 10.9 reflects the balance between the quantity and impact of their research, highlighting the consistent influence of remote sensing scientists. Similarly, the i10‐index, with an average value of 17.4, emphasizes the breadth of impactful contributions, representing the number of publications with at least 10 citations. Focusing on first‐author contributions, the analysis reveals that papers led by the researcher receive an average of 290 citations, though this figure shows considerable variability, with a standard deviation of 1122 (Table 1). This range suggests a spectrum of recognition for independent work, with some scientists achieving notable success while others emphasize collaborative projects. Collaboration appears to be a defining characteristic of the field, with 79% of citations stemming from co‐authored works, underscoring the highly cooperative nature of remote sensing research.

### 3.2 Keyword Network Visualization

**FIGURE 1:**
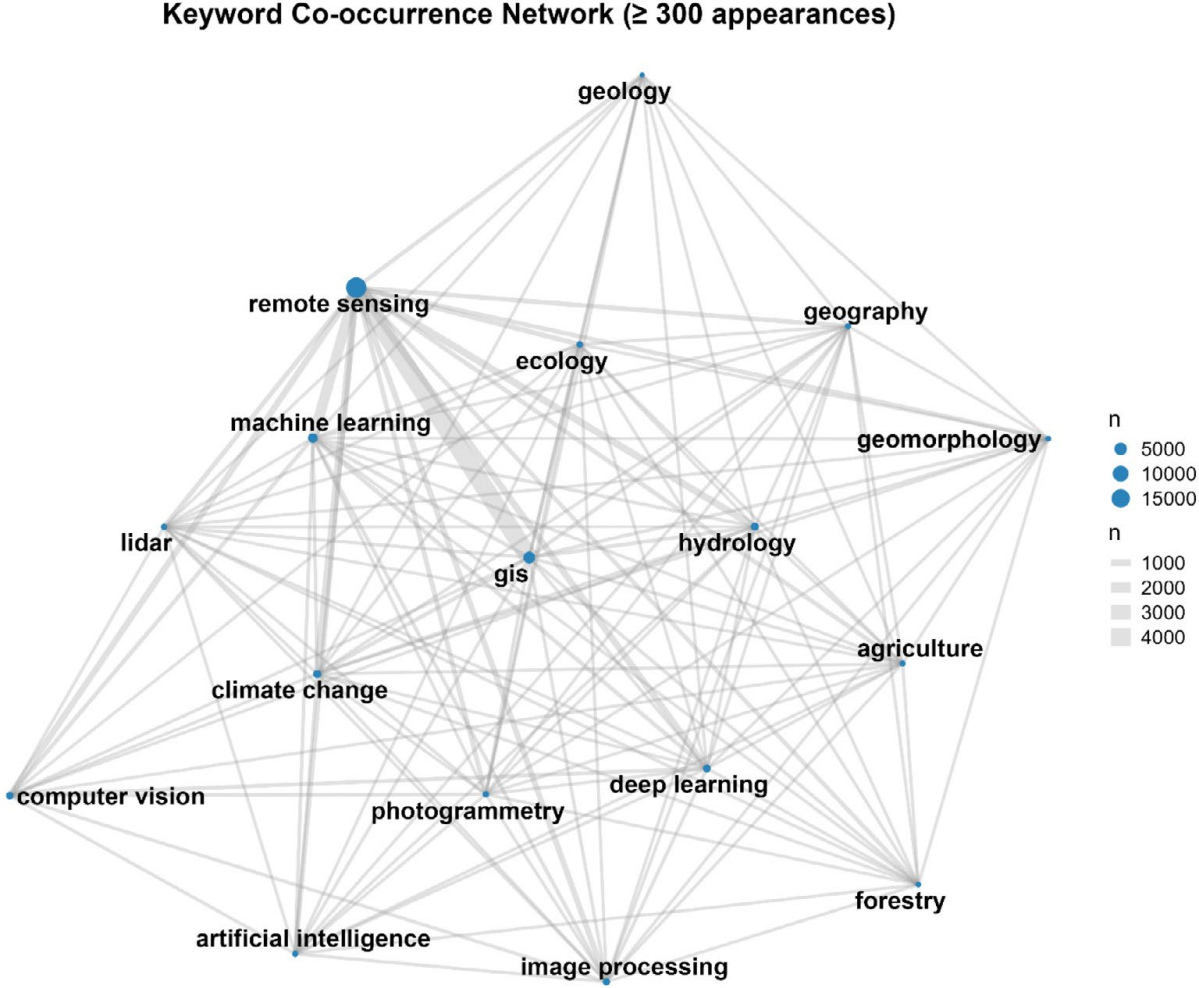
Network visualization of Google Scholar keywords with a minimum frequency of 300.

We gathered extensive data from 19,826 remote sensing scientists, capturing a total of 6076 unique keywords that reflect the diversity of their research interests and areas of expertise. Among these, the keyword “GIS” stood out as the most frequently mentioned, appearing 3890 times, highlighting its central role in remote sensing and ecological studies. Other commonly cited keywords included “machine learning” (1554 mentions), “hydrology” (1415 mentions), and “computer vision” (1256 mentions), emphasizing the field's integration of cutting‐edge technologies to study and preserve natural systems. Additional key themes included “deep learning” (1029 mentions), “image processing” (837 mentions), and “climate change” (759 mentions), showcasing the community's focus on leveraging technology to address global environmental challenges. To provide a clearer picture of the research landscape, we created a network visualization featuring keywords with a minimum of 300 mentions (Figure 1).

Each node in the network represents a unique keyword that appears in researchers' profiles with a frequency of at least 200 occurrences. The size of each node reflects how commonly that keyword is used across all profiles, with “remote sensing” expected to be the largest, given its presence in all entries. Edges between nodes indicate co‐occurrence of keywords within the same researcher profile, and the thickness of each edge represents the number of times that keyword pair appears together. This visualization reveals how researchers combine different thematic areas in their work, highlighting dominant research topics and subfields within the broader remote sensing community. For instance, strong connections between “remote sensing” and keywords like “climate change,” “land use,” or “machine learning” suggest major areas of interdisciplinary integration.

### 3.3 Trends in the Total Number of Papers and Citations

**FIGURE 2:**
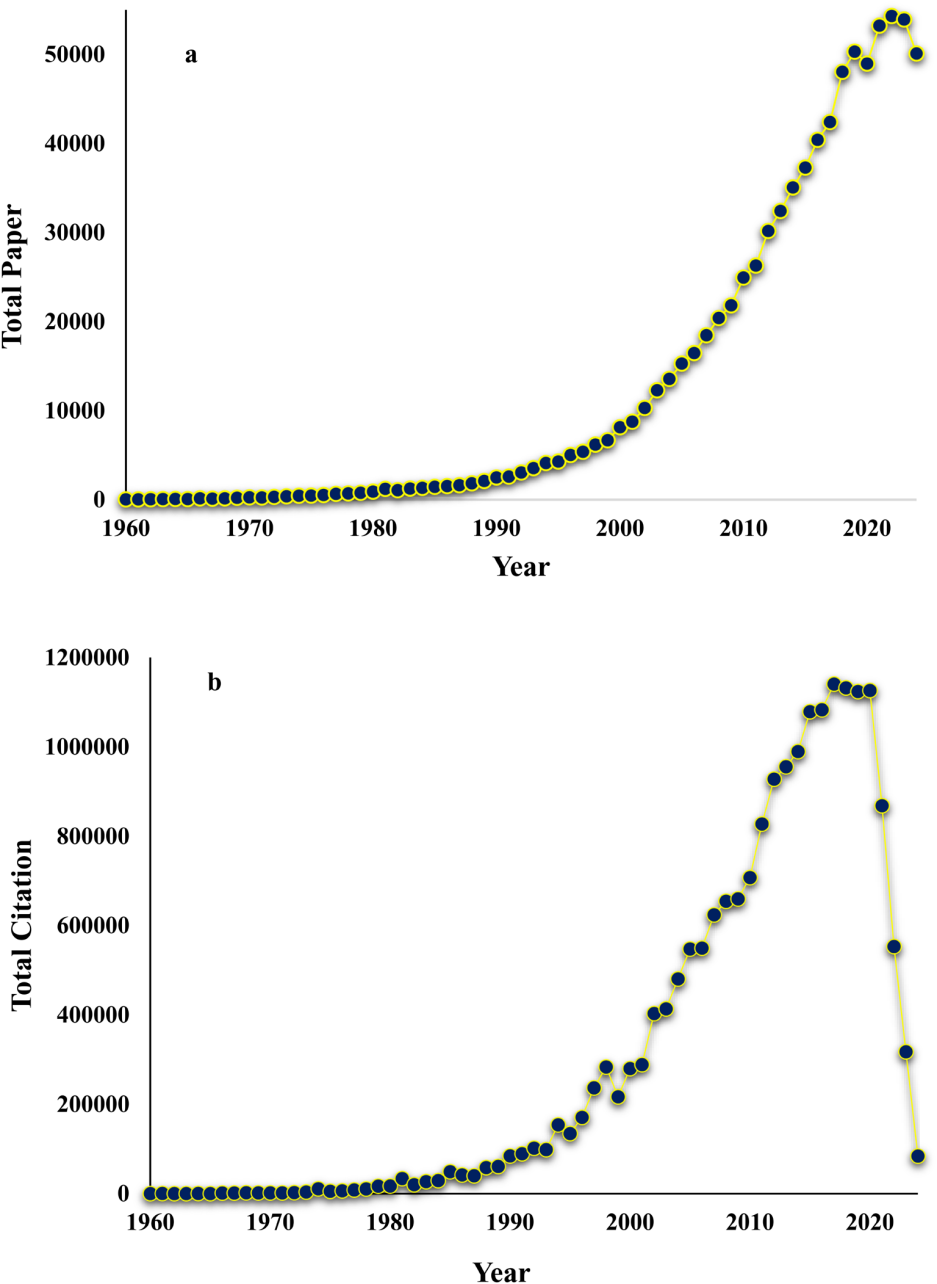
Trends in the total number of papers (a) and total citations (b) authored by 19,826 sensing scientists.

Figure 2a, the data illustrates the annual progression of scientific research output from 1960 to 2024, revealing significant trends and shifts in publication activity. Initially, research output was modest, with only 40 papers published in 1960. Through the 1970s and 1980s, the number of papers steadily increased, indicating a gradual expansion in scientific research. A notable surge occurred in the 1990s, with the annual output surpassing 8000 papers by 2000, signaling a period of rapid growth. This upward trajectory continued through the 2000s and early 2010s. The peak in scientific output was observed in 2022, with 54,304 papers published, marking the highest point in the timeline. Adjacent years, such as 2021 (53,226 papers) and 2023 (53,917 papers), also recorded similarly high outputs. However, a slight decline is evident in 2024, with 50,096 papers published.

Figure 2b illustrates the annual number of citations received by scientific publications from 1960 to 2024, offering a comprehensive view of the impact and recognition of research over time. In the early years, citation counts were relatively low, with 357 citations recorded in 1960. However, the numbers began to steadily rise in subsequent decades, reflecting the growing influence and dissemination of scientific work. By the late 1970s and early 1980s, citations reached notable levels, surpassing 10,000 annually, and continued to grow as research output expanded globally. The 1990s marked a period of significant growth in citations, with yearly counts exceeding 100,000 by the mid‐decade. This upward trend persisted into the 2000s and peaked in the mid‐2010s, with citations reaching their highest levels between 2015 and 2020. The year 2016, in particular, recorded over 1,083,016 citations, showcasing the cumulative impact of decades of scientific output. Recent years, however, show a notable decline in citation counts, with numbers dropping from over 1.1 million in 2020 to 317,585 in 2023 and just 84,389 in 2024.

**FIGURE 3:**
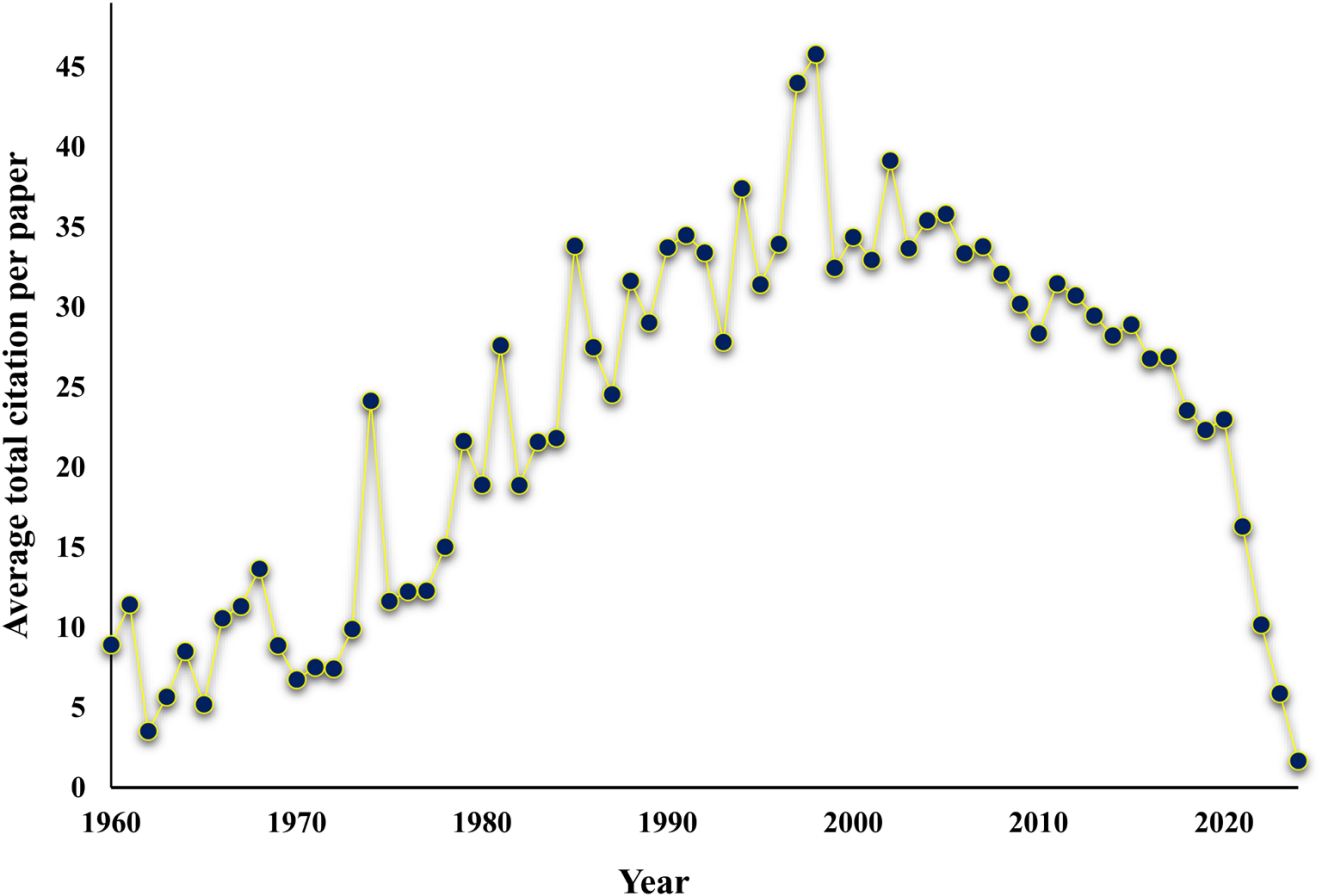
Trends in the average paper citation per year authored by 19,826 sensing scientists.

Figure 3 presents the average number of citations per paper per year from 1960 to 2024, shedding light on the relative impact of individual scientific publications. In 1960, the average number of citations per paper was relatively low, at approximately 10 citations per paper. However, this figure steadily increased over the next two decades, rising to over 27 citations per paper by 1970 and reaching 58 citations per paper by 1980. The period from 1980 to 2000 was marked by exponential growth in the average number of citations per paper. By 1990, the average had risen to 64 citations per paper, and by 2000, it exceeded 72 citations per paper. In the early 21st century (2000–2015), the average number of citations per paper peaked, often surpassing 95 citations in some years. However, after 2015, there was a noticeable decline in this metric, with the average dropping to 15 citations per paper by 2021 and further declining to approximately 1.4 citations per paper by 2024.

**FIGURE 4:**
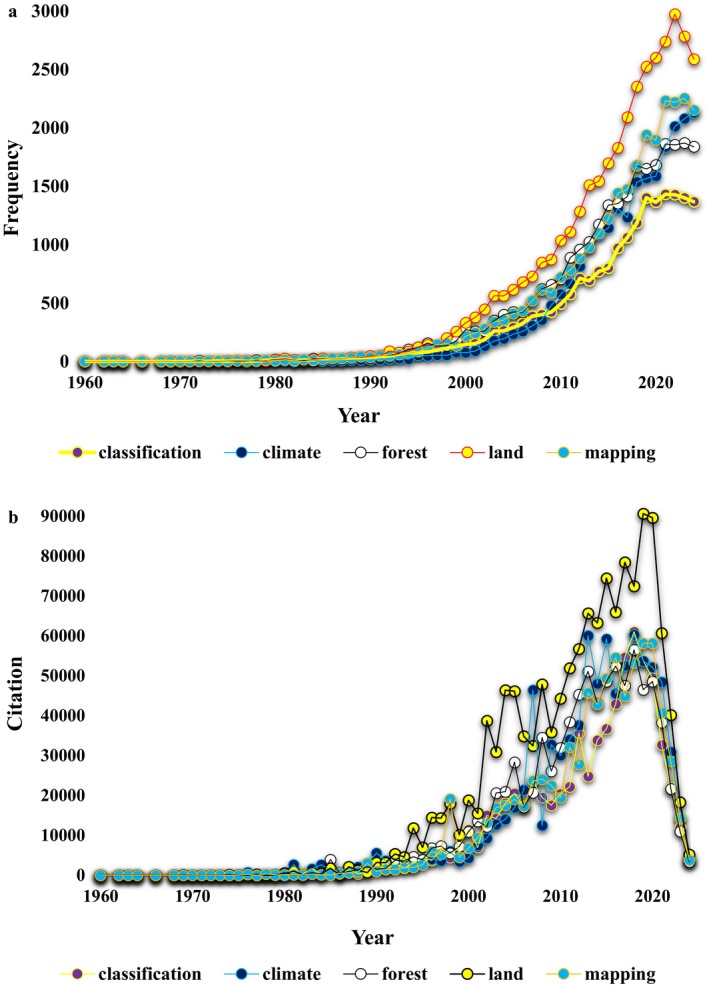
Trends in the total number of single keywords (a), related total citations (b), authored by 19,826 sensing scientists.

Figure 4 represents the trends in the total number of single keywords in paper titles authored by 19,826 sensing scientists (Figure 4a) and their corresponding citation counts from 1980 to 2024 (Figure 4b). The figure presents the annual trends in the total number of single keywords appearing in paper titles authored by remote sensing scientists, spanning from 1960 to 2024. The keywords tracked include “classification,” “climate,” “forest,” “land,” and “mapping,” reflecting the primary research areas within the field. Initially, from the 1960s to the 1980s, the occurrence of these keywords was minimal, with only sporadic mentions. For instance, in 1960, “climate” appeared once, while other keywords were either absent or had negligible occurrences. By the 1980s, there was a gradual increase, particularly in terms like “classification” and “land,” as research interest broadened.

The 1990s marked a turning point, with a notable rise in all keywords. By 1995, “forest” emerged as a prominent term with 121 mentions, reflecting a growing focus on forest ecosystems and their monitoring. Similarly, “mapping” and “land” saw significant increases, indicating the expanding application of remote sensing in land use and spatial analysis. The 2000s and 2010s witnessed a substantial surge across all keywords. For instance, “land” reached 1510 mentions by 2013, while “forest” and “climate” also showed rapid growth, surpassing 1000 mentions each. By 2020, “climate” reached a peak with 1587 mentions. In recent years, although the numbers remain high, slight fluctuations are evident. For example, “classification” peaked at 1432 mentions in 2021 before showing a slight decline in subsequent years. “Mapping” and “land” continue to dominate the titles, reflecting their enduring importance in remote sensing applications.

Figure 4b highlights the annual citations received for five major keywords from 1960 to 2024. In the early years (1960–1970), citations for all keywords were negligible, reflecting the nascent stage of remote sensing research. For instance, “climate” and “land” received zero citations in most years, and “mapping” first recorded a modest seven citations in 1966. By the late 1970s, citations began to increase, particularly for “classification” and “land,” as the field expanded its applications. The 1980s and 1990s marked a significant rise in citations for all keywords, signaling the broader adoption of remote sensing technologies. By 1995, “forest” citations had climbed to 3878, while “land” garnered 6990 citations, and “mapping” reached 2605. The year 1999 witnessed further growth, with “land” citations surpassing 10,000, underscoring its prominence in spatial analysis and land use studies.

The 2000s and 2010s represented a peak period for citations across all keywords. “Land” consistently emerged as the most cited term, reaching 74,432 citations in 2015, reflecting its central role in remote sensing research. Similarly, “classification” and “climate” saw substantial growth, with 61,050 and 60,294 citations, respectively, by 2018. “Forest” and “mapping” also gained prominence during this period, with “forest” citations peaking at 56,499 in 2018 and “mapping” reaching 53,232 the same year. Recent years (2020–2024) show a noticeable decline in citations for all keywords. By 2024, “classification” citations dropped to 3411, while “climate” fell to 3970, and “land” decreased to 5203. This decline could be attributed to shifts in research priorities or the maturation of previously dominant themes.

## 4 Discussion

This comprehensive bibliometric analysis revealed significant trends and patterns in the academic productivity, research impact, and collaborative networks of the global remote sensing (RS) community. Data encompassing 19,826 remote sensing scientists and 837,658 unique publications highlight a robust and dynamic research landscape. Collectively, these publications have garnered 19,852,700 citations, with an average of 1435 citations per researcher. However, a high standard deviation of 5768 underscores the variability in research impact, with some scientists achieving exceptional recognition while others exhibit moderate influence.

Key performance metrics, such as the average H‐index of 10.9 and an i10‐index of 17.4, demonstrate the consistent influence of remote sensing scientists. These metrics emphasize both the depth and breadth of impactful contributions within the field. First‐author papers received an average of 290 citations; though this figure varies widely, reflecting the diversity in independent research success and the prevalence of collaborative efforts. Notably, 79% of citations stem from co‐authored works, underscoring the highly cooperative nature of RS research. These findings align with Zhuang et al. (2013), who reported similar variability in citation metrics, emphasizing the influence of collaborative efforts in RS research. Additionally, Ju et al. (2024) found that international collaborations in mineral exploration contributed significantly to citation counts, a trend mirrored in our dataset. Rahimi and Jung (2025) also identified a total of 223 scientists active in pollination ecology, who together published 14,661 papers and amassed 1,570,139 citations. Interestingly, the majority of citations (67.8%) were from publications where these scientists were not listed as first authors. However, our findings on first‐author contributions provide a nuanced perspective, revealing a spectrum of recognition not emphasized in prior studies. This variability may indicate differing strategies among researchers, balancing independent versus collaborative work, and merits further exploration to identify factors influencing individual impact.

Keyword analysis sheds light on the thematic evolution of remote sensing studies. A total of 6076 unique keywords highlight the diversity of research interests, with “GIS” emerging as the most frequently mentioned keyword (3890 occurrences), followed by “machine learning” (1554), “hydrology” (1415), and “computer vision” (1256). These trends reflect the integration of advanced technologies and interdisciplinary approaches in remote sensing research. Other prominent topics, such as “deep learning” (1029), “image processing” (837), and “climate change” (759), further emphasize the growing focus on leveraging cutting‐edge tools to address global environmental challenges. These findings are consistent with Xu et al. (2022), who noted the widespread application of NDVI in monitoring vegetation dynamics and climate‐related phenomena. Similarly, Zhang et al. (2017) highlighted the adoption of machine learning techniques in RS, particularly for applications involving MODIS, Landsat, and SAR datasets. However, compared to Pham‐Duc et al. (2023), who emphasized groundwater and landslide research in Vietnam, our findings suggest a broader thematic focus, indicating diverse global applications of RS technologies.

The analysis of publication trends over time reveals exponential growth in RS research. From a modest 40 publications in 1960, the field expanded significantly through the 1990s, surpassing 8000 annual publications by 2000. This growth continued through the early 21st century, peaking in 2022 with 54,304 papers. Citations mirrored this trend, rising steadily from just 357 in 1960 to over 1.08 million in 2016. However, a noticeable decline in both publications and citations is observed in recent years, with 50,096 publications and 84,389 citations recorded in 2024. These fluctuations may indicate shifts in research priorities or the maturation of certain thematic areas. In a similar study, Rahimi and Jung (2025) identified a total of 223 scientists active in pollination ecology, who together published 14,661 papers and amassed 1,570,139 citations. The temporal trend revealed a steady rise in publication output beginning around 1974, reaching its peak in 2020, followed by a decline. Citation counts began to drop after 2010, potentially reflecting a move toward more specialized or fragmented areas of research.

This growth trajectory is consistent with findings from Zhang, Thenkabail, and Wang (2019), who reported exponential publication growth in RS journals. Similarly, Nduku et al. (2021) observed a steady rise in Earth Observation Systems research, attributed to advancements in satellite platforms and increased global collaboration. However, the decline in citations observed in our study diverges from the sustained citation impact reported by Balz (2022) for SAR‐related publications. This discrepancy may result from differences in topical focus, with niche studies in emerging fields gaining traction at the expense of broader thematic areas. The decline in citations could also reflect changes in scientific publishing practices, as noted by Sebastiá‐Frasquet et al. (2020), who discussed the impact of preprints and open‐access platforms on traditional citation metrics.

The use of Google Scholar as the primary data source shaped several of the key patterns observed in this study. For example, the high average citation count (1435 per researcher) and elevated h‐ and i10‐index values may partially reflect the inclusion of a broader range of document types, such as preprints, theses, and non‐peer‐reviewed materials, which are commonly indexed by Google Scholar. Similarly, the large proportion of citations from co‐authored papers (79%) may be influenced by the way Google Scholar attributes publications across collaborative networks, sometimes more inclusively than curated databases. The wide thematic spread revealed by keyword analysis—spanning GIS, machine learning, hydrology, and climate—also benefits from Google Scholar's ability to capture interdisciplinary work that might otherwise be excluded under stricter indexing criteria. While these patterns may introduce certain biases, they also offer a more comprehensive view of author‐level productivity and collaboration in remote sensing, particularly useful in tracking the full academic footprint of researchers in a globally dispersed field.

## 5 Limitations

While this study offers valuable insights into the global landscape of remote sensing research, several limitations must be acknowledged. First, our analysis is solely based on data extracted from Google Scholar profiles, which, although comprehensive, introduces certain biases. Google Scholar tends to include a wide range of document types, including non‐peer‐reviewed materials such as theses, reports, and preprints, which may inflate citation counts or obscure the scientific rigor of included publications. Additionally, it lacks the standardization and curation of databases such as Web of Science or Scopus, limiting cross‐validation and potentially affecting the reliability of citation‐based metrics.

Another important limitation lies in the level of geographic granularity. Our analysis could not delve into more localized patterns at the regional or institutional level. This omission may overlook critical contextual factors—such as funding environments, infrastructure, or local academic networks—that drive or constrain research activity in remote sensing. Furthermore, while we observed a recent decline in citation trends, our study does not fully investigate the underlying causes of this pattern. Factors such as the increasing prevalence of open‐access publishing, the rise of preprint culture, changes in citation behavior, or research fragmentation may contribute to these trends and warrant further exploration. In terms of thematic analysis, our reliance on author‐provided keywords may lead to an oversimplified view of the field. Emerging or interdisciplinary topics that are not captured through frequently used keywords could be underrepresented, limiting our ability to detect nuanced or novel research directions. A more refined topic‐modeling or content‐based approach could help reveal hidden thematic structures within the literature.

Moreover, this study focuses exclusively on quantitative metrics—such as publication counts, citations, and h‐index values—without evaluating the qualitative dimensions of research, such as scientific innovation, policy impact, or practical applications. This limits the extent to which our findings can inform strategic planning or research prioritization beyond academic metrics. A further methodological constraint arises from our profile‐based sampling approach. By targeting researchers explicitly identifying with “remote sensing” in their Google Scholar profiles, we captured the entirety of their academic outputs, including works outside the strict boundaries of the field. While this provides a broad view of these scholars' academic impact, it may also skew the representation of remote sensing‐specific literature. Conversely, researchers making meaningful contributions to remote sensing without labeling themselves as such may have been inadvertently excluded.

## 6 Conclusion

This study highlights the transformative journey of remote sensing research over the past six decades, reflecting its emergence from a niche scientific area to a broad, interdisciplinary field of critical global importance. The growth in scholarly output and collaboration underscores the increasing recognition of remote sensing as an indispensable tool for addressing complex environmental, social, and technological challenges. However, the observed recent declines in citation rates and publication growth suggest that the field is entering a period of reflection and realignment. Such trends may reflect evolving research priorities, the diversification and specialization of subfields, or changing scholarly communication practices, including the rise of preprints and open‐access models. These dynamics indicate that the field must continually adapt to remain at the forefront of scientific inquiry and societal relevance.

Our findings on the dominant themes and collaborative nature of remote sensing emphasize the ongoing integration of cutting‐edge technologies like machine learning and AI, while also pointing to emerging challenges in sustaining innovation and impact. Importantly, this work reveals that measuring academic influence solely through quantitative metrics may mask underlying complexities such as differences in career stage, geographic disparities, and institutional contexts. The variability in individual researchers' citation impact suggests diverse career trajectories and strategic approaches to collaboration and leadership. Future studies would benefit from incorporating qualitative assessments and longitudinal career data to more fully understand how knowledge production and dissemination evolve within the community.

## Author Contributions

**Ehsan Rahimi:** conceptualization (equal), data curation (equal), formal analysis (equal), investigation (equal), methodology (equal), software (equal), writing – original draft (equal). **Chuleui Jung:** conceptualization (equal), formal analysis (equal), funding acquisition (lead), investigation (lead), project administration (lead), resources (lead), supervision (lead), validation (lead), writing – review and editing (lead).

## Conflicts of Interest

The authors declare no conflicts of interest.

## Data Availability

Supporting Information is available at https://github.com/ehsanrahimi666/Remote‐sensing.git.

## References

[ece371952-bib-0001] Aleixandre‐Benavent, R. , J. L. Aleixandre‐Tudó , L. Castelló‐Cogollos , and J. L. Aleixandre . 2018. “Trends in Global Research in Deforestation. A Bibliometric Analysis.” Land Use Policy 72: 293–302.

[ece371952-bib-0002] Badaluddin, N. A. , M. Lion , S. M. Razali , and S. I. Khalit . 2021. “Bibliometric Analysis of Global Trends on Soil Moisture Assessment Using the Remote Sensing Research Study From 2000 to 2020.” Water, Air, and Soil Pollution 232: 271.

[ece371952-bib-0003] Balz, T. 2022. “Scientometric Full‐Text Analysis of Papers Published in Remote Sensing Between 2009 and 2021.” Remote Sensing 14: 4285.

[ece371952-bib-0004] Bjurström, A. , and M. Polk . 2011. “Climate Change and Interdisciplinarity: A Co‐Citation Analysis of IPCC Third Assessment Report.” Scientometrics 87: 525–550.

[ece371952-bib-0005] Borthakur, A. , and P. Singh . 2018. “Global Research Trends in ‘Ecology’: A Scientometric Analysis.” Tropical Ecology 59: 431–443.

[ece371952-bib-0006] Chen, S. , J. Chen , C. Jiang , et al. 2022. “Trends in Research on Forest Ecosystem Services in the Most Recent 20 Years: A Bibliometric Analysis.” Forests 13: 1087.

[ece371952-bib-0007] Chen, X. , F. Yuan , S. T. Ata‐Ul‐Karim , et al. 2025. “A Bibliometric Analysis of Research on Remote Sensing‐Based Monitoring of Soil Organic Matter Conducted Between 2003 and 2023.” Artificial Intelligence in Agriculture 15: 26–38.

[ece371952-bib-0008] Collins, M. K. , S. B. Magle , and T. Gallo . 2021. “Global Trends in Urban Wildlife Ecology and Conservation.” Biological Conservation 261: 109236.

[ece371952-bib-0009] Costa, D. P. , S. M. Herrmann , R. N. Vasconcelos , et al. 2023. “Bibliometric Analysis of Land Degradation Studies in Drylands Using Remote Sensing Data: A 40‐Year Review.” Land 12: 1721.

[ece371952-bib-0010] Das, B. K. , M. Pandya , S. Chaudhari , A. Bhatt , and D. Trivedi . 2021. “Global Research Trends and Network Visualization on Climate Action: A Bibliometric Study.” Library Philosophy and Practice (e‐Journal) 68: 152–169. https://digitalcommons.unl.edu/libphilprac/5818.

[ece371952-bib-0011] Dash, J. , and B. O. Ogutu . 2016. “Recent Advances in Space‐Borne Optical Remote Sensing Systems for Monitoring Global Terrestrial Ecosystems.” Progress in Physical Geography 40: 322–351.

[ece371952-bib-0012] Deng, J. , Y. Zhang , B. Qin , X. Yao , and Y. Deng . 2017. “Trends of Publications Related to Climate Change and Lake Research From 1991 to 2015.” Journal of Limnology 76: 439–450.

[ece371952-bib-0013] Duan, P. , Y. Wang , and P. Yin . 2020. “Remote Sensing Applications in Monitoring of Protected Areas: A Bibliometric Analysis.” Remote Sensing 12: 772.

[ece371952-bib-0014] Fan, J. , G. Liu , Z. Xia , and S. Cai . 2022. “A Bibliometric Analysis of Climate Change Risk Perception: Hot Spots, Trends and Improvements.” Frontiers in Environmental Science 10: 917469.

[ece371952-bib-0015] Gangahagedara, R. , S. Subasinghe , M. Lankathilake , W. Athukorala , and I. Gamage . 2021. “Ecosystem Services Research Trends: A Bibliometric Analysis From 2000–2020.” Ecologies 2: 366–379.

[ece371952-bib-0016] Haunschild, R. , L. Bornmann , and W. Marx . 2016. “Climate Change Research in View of Bibliometrics.” PLoS One 11: e0160393. 27472663 10.1371/journal.pone.0160393 PMC4966958

[ece371952-bib-0017] Holyoak, M. , R. Casagrandi , R. Nathan , E. Revilla , and O. Spiegel . 2008. “Trends and Missing Parts in the Study of Movement Ecology.” Proceedings of the National Academy of Sciences 105: 19060–19065. 10.1073/pnas.0800483105 PMC2614715 19060194

[ece371952-bib-0018] Hussain, A. 2017. “A Scientometric Analysis of the ‘Journal of King Saud University‐Computer and Information Sciences’.” Library Philosophy and Practice (e‐Journal) 1528. May 4. https://digitalcommons.unl.edu/libphilprac/1528.

[ece371952-bib-0019] Jiang, Y. , C. Guo , F. Su , et al. 2024. “Climate Warming Effects on Temperature Structure in Lentic Waters: A Bibliometric Analysis From the Recent 20 Years.” Ecological Indicators 167: 112740.

[ece371952-bib-0020] Ju, L. , Y. Liu , S. Liu , Q. Xiang , W. Hu , and P. Yu . 2024. “Bibliometric Analysis of Global Trends and Characteristics of Remote Sensing for Mineral Exploration in the Early 21st Century.” All Earth 36: 1–17.

[ece371952-bib-0021] Khorram, S. , C. F. van der Wiele , F. H. Koch , S. A. Nelson , and M. D. Potts . 2016. “Future Trends in Remote Sensing.” In Principles of Applied Remote Sensing, 277–285. Springer.

[ece371952-bib-0022] Li, C. , H. Yao , Z. Li , et al. 2024. “A Bibliometric Analysis of Global Research on Climate Change and Agriculture From 1985 to 2023.” Agronomy 14: 2729.

[ece371952-bib-0023] Li, J. , X. Zheng , and C. Zhang . 2021. “Retrospective Research on the Interactions Between Land‐Cover Change and Global Warming Using Bibliometrics During 1991–2018.” Environmental Earth Sciences 80: 573.

[ece371952-bib-0024] Li, L. , X. Xin , J. Zhao , et al. 2023. “Remote Sensing Monitoring and Assessment of Global Vegetation Status and Changes During 2016–2020.” Sensors 23: 8452. 37896545 10.3390/s23208452 PMC10611270

[ece371952-bib-0025] Li, T. , L. Cui , Z. Xu , et al. 2021. “Quantitative Analysis of the Research Trends and Areas in Grassland Remote Sensing: A Scientometrics Analysis of Web of Science From 1980 to 2020.” Remote Sensing 13: 1279.

[ece371952-bib-0026] Liang, B. , G. Shi , Z. Sun , H. Babul , and M. Zhou . 2023. “Evolution Trend and Hot Topic Measurement of Climate Migration Research Under the Influence of Climate Change.” Frontiers in Ecology and Evolution 11: 1118037.

[ece371952-bib-0027] Lindawati, A. , and Meiryani . 2024. “A Bibliometric Analysis on the Research Trends of Global Climate Change and Future Directions.” Cogent Business & Management 11: 2325112.

[ece371952-bib-0028] Liu, W. , J. Wang , C. Li , B. Chen , and Y. Sun . 2019. “Using Bibliometric Analysis to Understand the Recent Progress in Agroecosystem Services Research.” Ecological Economics 156: 293–305.

[ece371952-bib-0029] Marx, W. , R. Haunschild , and L. Bornmann . 2017. “Climate Change and Viticulture—A Quantitative Analysis of a Highly Dynamic Research Field.” Vitis 56: 35–43.

[ece371952-bib-0030] Marx, W. , R. Haunschild , and L. Bornmann . 2021. “Heat Waves: A Hot Topic in Climate Change Research.” Theoretical and Applied Climatology 146: 781–800. 34493886 10.1007/s00704-021-03758-y PMC8414451

[ece371952-bib-0031] Nalau, J. , and B. Verrall . 2021. “Mapping the Evolution and Current Trends in Climate Change Adaptation Science.” Climate Risk Management 32: 100290.

[ece371952-bib-0032] Nduku, L. , A. M. Kalumba , C. Munghemezulu , et al. 2021. “Earth Observation Systems and Pasture Modeling: A Bibliometric Trend Analysis.” ISPRS International Journal of Geo‐Information 10: 793.

[ece371952-bib-0033] Nduku, L. , C. Munghemezulu , Z. Mashaba‐Munghemezulu , et al. 2023. “Global Research Trends for Unmanned Aerial Vehicle Remote Sensing Application in Wheat Crop Monitoring.” Geomatics 3: 115–136.

[ece371952-bib-0034] Pham‐Duc, B. , H. Nguyen , C. Le Minh , L. H. Khanh , and T. Trung . 2020. “A Bibliometric and Content Analysis of Articles in Remote Sensing From Vietnam Indexed in Scopus for the 2000–2019 Period.” Serials Review 46: 275–285.

[ece371952-bib-0035] Pham‐Duc, B. , H. Nguyen , H. Phan , and Q. Tran‐Anh . 2023. “Trends and Applications of Google Earth Engine in Remote Sensing and Earth Science Research: A Bibliometric Analysis Using Scopus Database.” Earth Science Informatics 16: 2355–2371.

[ece371952-bib-0036] Pius Awhari, D. , M. H. B. Jamal , M. K. I. Muhammad , and S. Shahid . 2024. “Bibliometric Analysis of Global Climate Change and Agricultural Production: Trends, Gaps and Future Directions.” Irrigation and Drainage 73: 1615–1632.

[ece371952-bib-0037] Rahimi, E. , P. Dong , and C. Jung . 2025. “Global NDVI‐LST Correlation: Temporal and Spatial Patterns From 2000 to 2024.” Environments 12: 67.

[ece371952-bib-0038] Rahimi, E. , and C. Jung . 2025. “Trends in Pollination Scientists' Research: A Comprehensive Analysis in Citations and Research Topics.” Ecology and Evolution 15: e71215. 40336547 10.1002/ece3.71215 PMC12058208

[ece371952-bib-0039] Santos, S. M. B. D. , A. Bento‐Gonçalves , and A. Vieira . 2021. “Research on Wildfires and Remote Sensing in the Last Three Decades: A Bibliometric Analysis.” Forests 12: 604.

[ece371952-bib-0040] Sebastiá‐Frasquet, M.‐T. , J.‐A. Aguilar‐Maldonado , I. Herrero‐Durá , E. Santamaría‐del‐Ángel , S. Morell‐Monzó , and J. Estornell . 2020. “Advances in the Monitoring of Algal Blooms by Remote Sensing: A Bibliometric Analysis.” Applied Sciences 10: 7877.

[ece371952-bib-0041] Setiawan, D. , I. P. Rahmawati , and A. Santoso . 2023. “A Bibliometric Analysis of Evolving Trends in Climate Change and Accounting Research.” Cogent Business & Management 10: 2267233.

[ece371952-bib-0042] Shi, X. , J. Zhang , J. Lu , et al. 2024. “Global Trends and Innovations in Forest Ecological Compensation: An Interdisciplinary Analysis.” Forests 15: 631.

[ece371952-bib-0043] Sun, S. , Y. Jiang , and S. Zheng . 2020. “Research on Ecological Infrastructure From 1990 to 2018: A Bibliometric Analysis.” Sustainability 12: 2304.

[ece371952-bib-0044] Sweileh, W. M. 2020. “Bibliometric Analysis of Peer‐Reviewed Literature on Food Security in the Context of Climate Change From 1980 to 2019.” Agriculture & Food Security 9: 1–15.

[ece371952-bib-0045] Tasneem, S. , and M. N. Ahsan . 2024. “A Bibliometric Analysis on Mangrove Ecosystem Services: Past Trends and Emerging Interests.” Ocean and Coastal Management 256: 107276.

[ece371952-bib-0046] Valjarević, A. , T. Djekić , V. Stevanović , R. Ivanović , and B. Jandziković . 2018. “GIS Numerical and Remote Sensing Analyses of Forest Changes in the Toplica Region for the Period of 1953–2013.” Applied Geography 92: 131–139.

[ece371952-bib-0047] Velastegui‐Montoya, A. , N. Montalván‐Burbano , P. Carrión‐Mero , H. Rivera‐Torres , L. Sadeck , and M. Adami . 2023. “Google Earth Engine: A Global Analysis and Future Trends.” Remote Sensing 15: 3675.

[ece371952-bib-0048] Vihervaara, P. , M. Rönkä , and M. Walls . 2010. “Trends in Ecosystem Service Research: Early Steps and Current Drivers.” Ambio 39: 314–324. 20799681 10.1007/s13280-010-0048-x PMC3357705

[ece371952-bib-0049] Wang, B. , Q. Zhang , and F. Cui . 2021. “Scientific Research on Ecosystem Services and Human Well‐Being: A Bibliometric Analysis.” Ecological Indicators 125: 107449.

[ece371952-bib-0050] Wei, X. , W. Song , Y. Shao , and X. Cai . 2022. “Progress of Ecological Restoration Research Based on Bibliometric Analysis.” International Journal of Environmental Research and Public Health 20: 520. 36612842 10.3390/ijerph20010520 PMC9819557

[ece371952-bib-0051] Wentz, E. A. , S. Anderson , M. Fragkias , et al. 2014. “Supporting Global Environmental Change Research: A Review of Trends and Knowledge Gaps in Urban Remote Sensing.” Remote Sensing 6: 3879–3905.

[ece371952-bib-0052] Xu, Y. , Y. Yang , X. Chen , and Y. Liu . 2022. “Bibliometric Analysis of Global NDVI Research Trends From 1985 to 2021.” Remote Sensing 14: 3967.

[ece371952-bib-0053] Zeng, Y. , J. Zhang , and R. Niu . 2015. “Research Status and Development Trend of Remote Sensing in China Using Bibliometric Analysis.” International Archives of the Photogrammetry, Remote Sensing and Spatial Information Sciences 40: 203–208.

[ece371952-bib-0054] Zhang, H. , M. Huang , X. Qing , G. Li , and C. Tian . 2017. “Bibliometric Analysis of Global Remote Sensing Research During 2010–2015.” ISPRS International Journal of Geo‐Information 6: 332.

[ece371952-bib-0055] Zhang, L. , M.‐H. Wang , J. Hu , and Y.‐S. Ho . 2010. “A Review of Published Wetland Research, 1991–2008: Ecological Engineering and Ecosystem Restoration.” Ecological Engineering 36: 973–980.

[ece371952-bib-0056] Zhang, X. , R. C. Estoque , H. Xie , Y. Murayama , and M. Ranagalage . 2019. “Bibliometric Analysis of Highly Cited Articles on Ecosystem Services.” PLoS One 14: e0210707. 30742632 10.1371/journal.pone.0210707 PMC6370190

[ece371952-bib-0057] Zhang, Y. , P. S. Thenkabail , and P. Wang . 2019. “A Bibliometric Profile of the Remote Sensing Open Access Journal Published by MDPI Between 2009 and 2018.” Remote Sensing 11: 91.

[ece371952-bib-0058] Zheng, J. , L. Wang , and C. Li . 2023. “Trends and Hotspots in Riparian Restoration Research: A Global Bibliometric Analysis During 1990–2022.” Forests 14: 2205.

[ece371952-bib-0059] Zhuang, Y. , X. Liu , T. Nguyen , Q. He , and S. Hong . 2013. “Global Remote Sensing Research Trends During 1991–2010: A Bibliometric Analysis.” Scientometrics 96: 203–219.

